# Supplementary material for: Spatiotemporal clustering, climate periodicity, and social-ecological risk factors for dengue during an outbreak in Machala, Ecuador, in 2010
Source: BMC Infect Dis. 2014 Nov 25;14:610. doi: 10.1186/s12879-014-0610-4 (PMC4264610; doi:10.1186/s12879-014-0610-4)
Supplement: Supplementary file 1 — Additional file 1: Table S1.: Spanish dictionary of census variables evaluated in the multivariate model to predict the presence of dengue. (DOCX 18 KB) [file 12879_2014_610_MOESM1_ESM.docx]

| **Table S1. Spanish dictionary of census variables evaluated in the multivariate model to predict the presence of dengue.** | | | | |
| --- | --- | --- | --- | --- |
| **Censo*** | **Variable original** | **Recodificacion** | **Filtro** | **Descripcion** |
| P | P03 Cuantos años cumplidos tiene | n/a |  | Promedio del edad de la casa |
| P | P03 Cuantos años cumplidos tiene | n/a | P02 = 1 | Promedio del edad del jefe de la casa |
| P |  |  |  | Personas por hogar |
| P | P23 Cuales el nivel de instrucción más alto al que asiste o asistió | P23 <= 4 | P02 = 1 | Porcentaje de hogares donde el jefe del hogar tiene educación primaria o mes |
| P | P16 Como se identifica según su cultura y costumbres | P16==2 \| P16==3 \| P16==4 |  | Porcentaje de la población que son Afro-Ecuatoriano, mulato o Negro |
| P | P27 Que hizo la semana pasado | P27 == 7 | P02 = 1 | Porcentaje de hogares con el jefe del hogar desempleado |
| P | P01 Cual es el sexo | P01 == 2 | P02 = 1 | Porcentaje de hogares con una mujer como jefa del hogar |
| V | V02 (estado del techo), V04 (estado de las paredes), V06 (estado del piso) | (sum (V02, V04,V06)-3)/6 | TOTPER>0 | Índice de condición del hogar normalizado (compuesto de 3 parametros, 0 -1, donde 1 = peor) |
| V | TOTPER Total de personas de la vivienda | TOTPER==0 |  | Porcentaje de hogares desocupados |
| V | PERDOR Numero de personas pordormitorio | PERDOR >= 4 |  | Porcentaje de hogares con más de 4 personas por dormitorio |
| V | V13 Principalmente como elimina la basura | V13 > 1 | TOTPER>0 | Porcentaje de hogares que eliminan la basura por carro recolector |
| V | V08 El agua que recibe la vivienda es: | V08 > 1 | TOTPER>0 | Porcentaje de hogares SIN agua por tubería dentro de la casa |
| V | V09 El servicio higiénico o escusado de la vivienda es | V09 > 1 | TOTPER>0 | Porcentaje de hogares SIN conexión al red público de alcantarillado |
| V | Vap: vía de acceso principal a la vivienda | VAP > 2 | TOTPER>0 | Porcentaje de hogares que tienen calle o carretera adoquinada, pavimentada o de concreto |
| V | V16 Cuantos grupos de personas(hogares) duermen en su vivienda y cocinan los alimentos por separado incluya su hogar | V16 == 2 | TOTPER>0 | Porcentaje de hogares que comparten su casa con más de otro hogar |
| V | VIVREM viviendas con remesas | VIVREM == 1 | TOTPER>0 | Porcentaje de hogares que reciben remesas |
| V | TOTEMI Total de migrantes | TOTEMI > 1 | TOTPER>0 | Porcentaje de hogares que tienen emigrantes |
| H | H06 Principalmente, el agua que toman los miembros del hogar | H06 < 5 |  | Porcentaje de hogares que toman agua de la llave |
| H | H15 La vivienda que ocupa este hogares… | H15==6 |  | Porcentaje de hogares que alquilan |

*P = censo de población, H = censo de hogar, V = censo de vivienda
