# Supplementary figures and images for: Spatiotemporal clustering, climate periodicity, and social-ecological risk factors for dengue during an outbreak in Machala, Ecuador, in 2010
Source: BMC Infect Dis. 2014 Nov 25;14:610. doi: 10.1186/s12879-014-0610-4 (PMC4264610; doi:10.1186/s12879-014-0610-4)

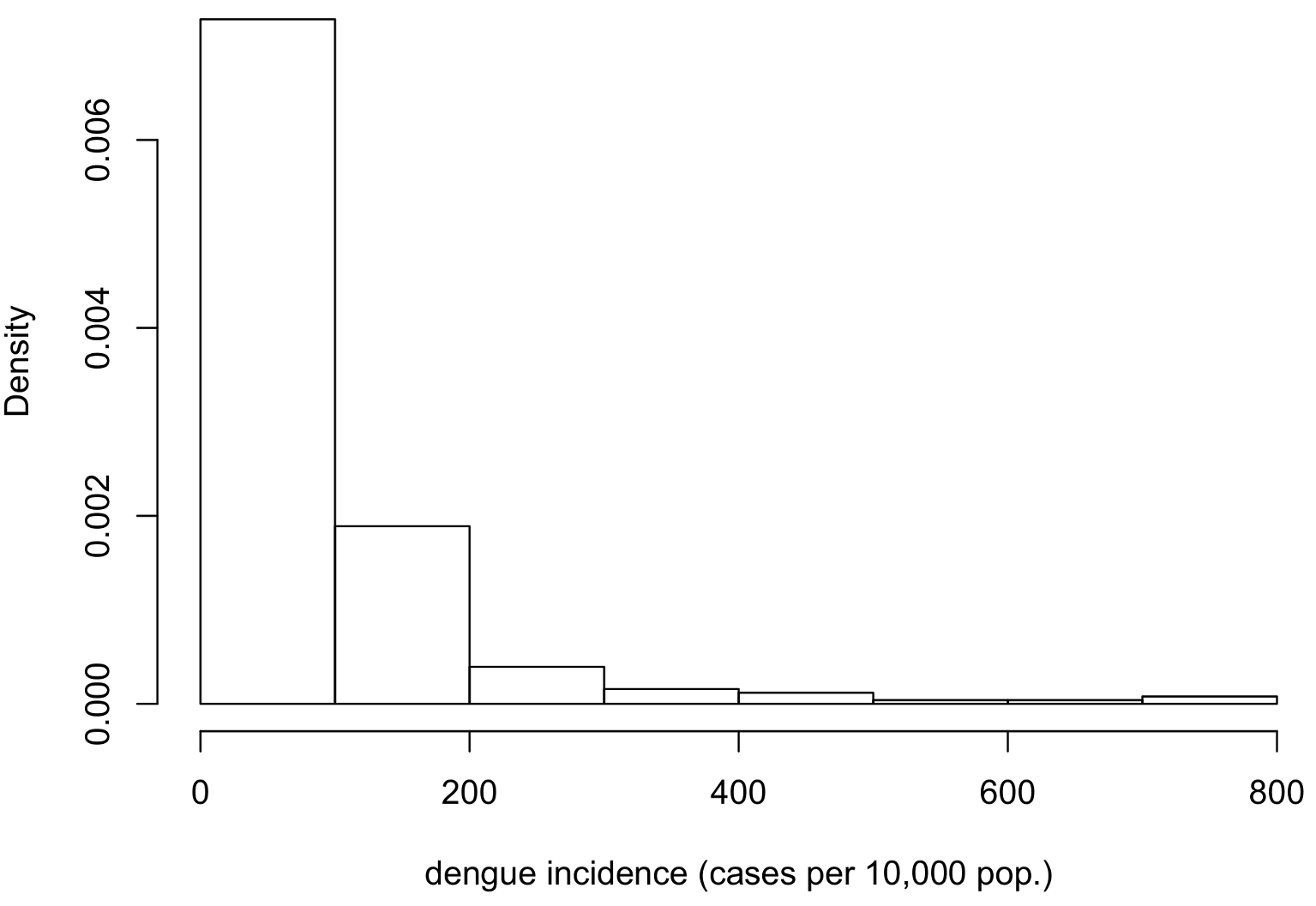

Supplement: Supplementary file 2 — Additional file 2: Figure S1.: Histogram showing the density distribution of neighborhood dengue incidence in Machala, 2010 (n = 253). (TIFF 89 KB) [file 12879_2014_610_MOESM2_ESM.tiff]

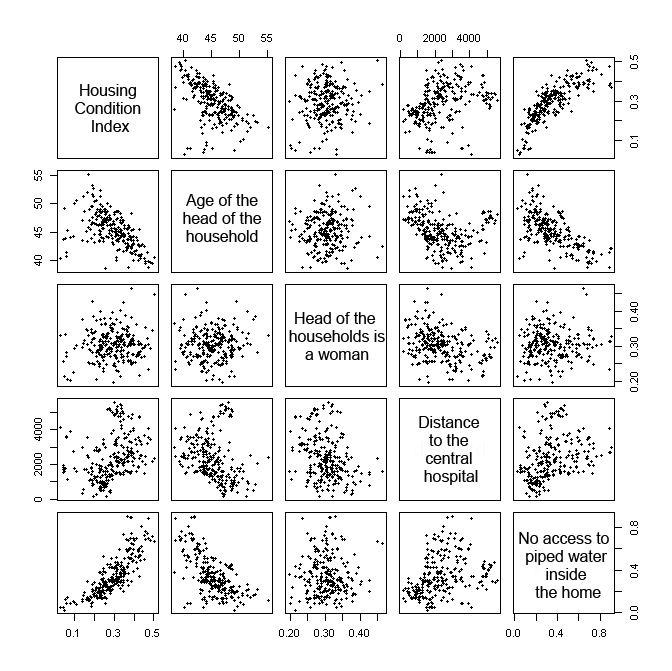

Supplement: Supplementary file 3 — Additional file 3: Figure S2.: Scatter matrix of parameters included in the top logistic regression model to predict the presence of dengue in neighborhoods in Machala in 2010. (TIFF 77 KB) [file 12879_2014_610_MOESM3_ESM.tiff]

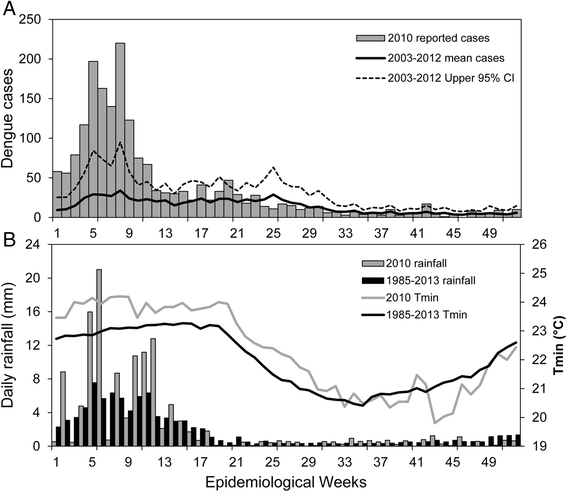

Supplement: Supplementary file 5 — Authors’ original file for figure 1 [file 12879_2014_610_MOESM5_ESM.gif]

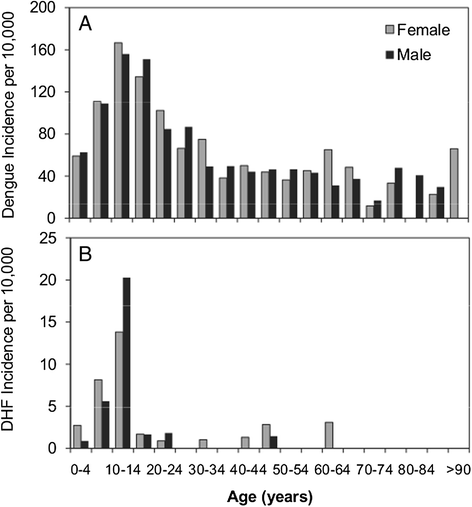

Supplement: Supplementary file 6 — Authors’ original file for figure 2 [file 12879_2014_610_MOESM6_ESM.gif]

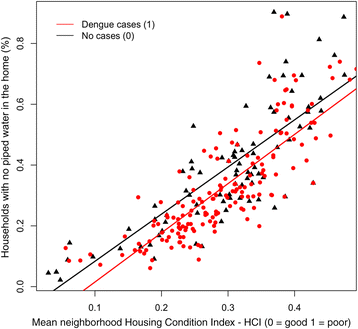

Supplement: Supplementary file 7 — Authors’ original file for figure 3 [file 12879_2014_610_MOESM7_ESM.gif]

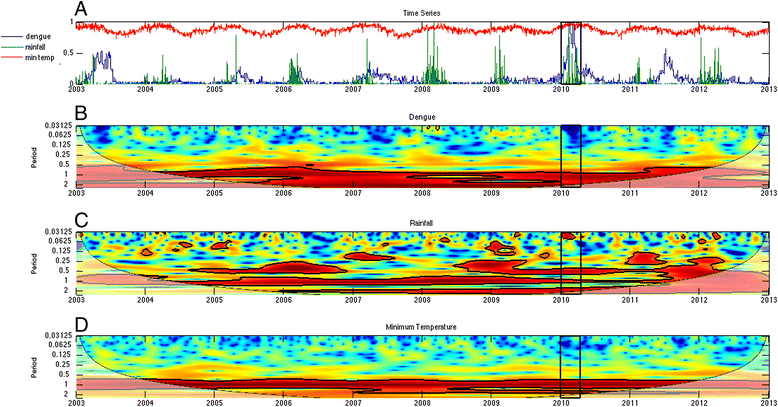

Supplement: Supplementary file 8 — Authors’ original file for figure 4 [file 12879_2014_610_MOESM8_ESM.gif]

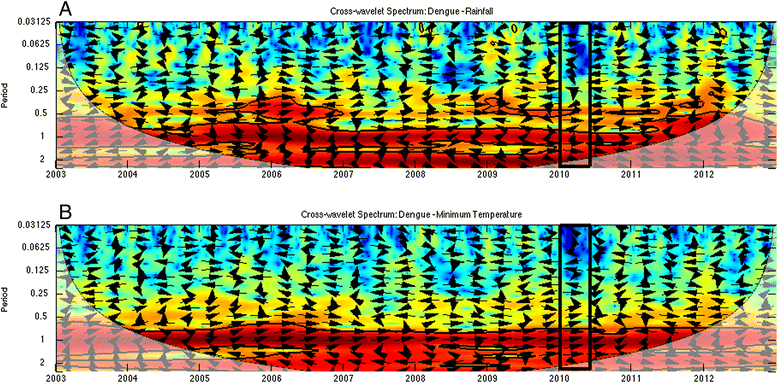

Supplement: Supplementary file 9 — Authors’ original file for figure 5 [file 12879_2014_610_MOESM9_ESM.gif]

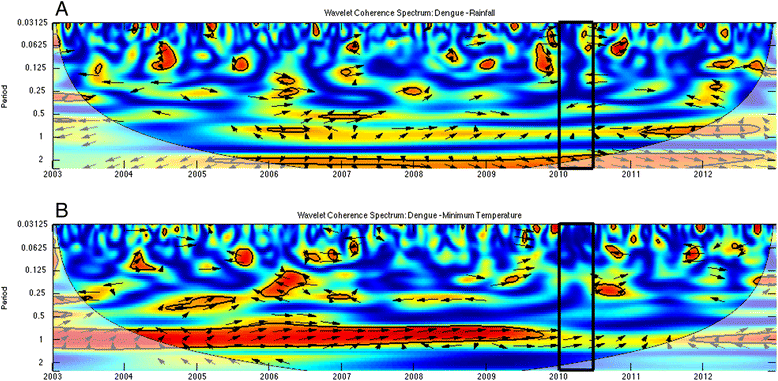

Supplement: Supplementary file 10 — Authors’ original file for figure 6 [file 12879_2014_610_MOESM10_ESM.gif]

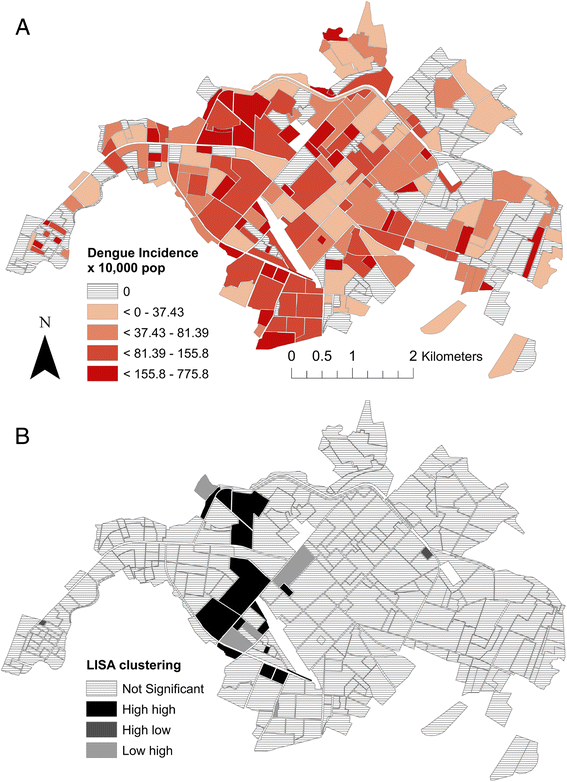

Supplement: Supplementary file 11 — Authors’ original file for figure 7 [file 12879_2014_610_MOESM11_ESM.gif]

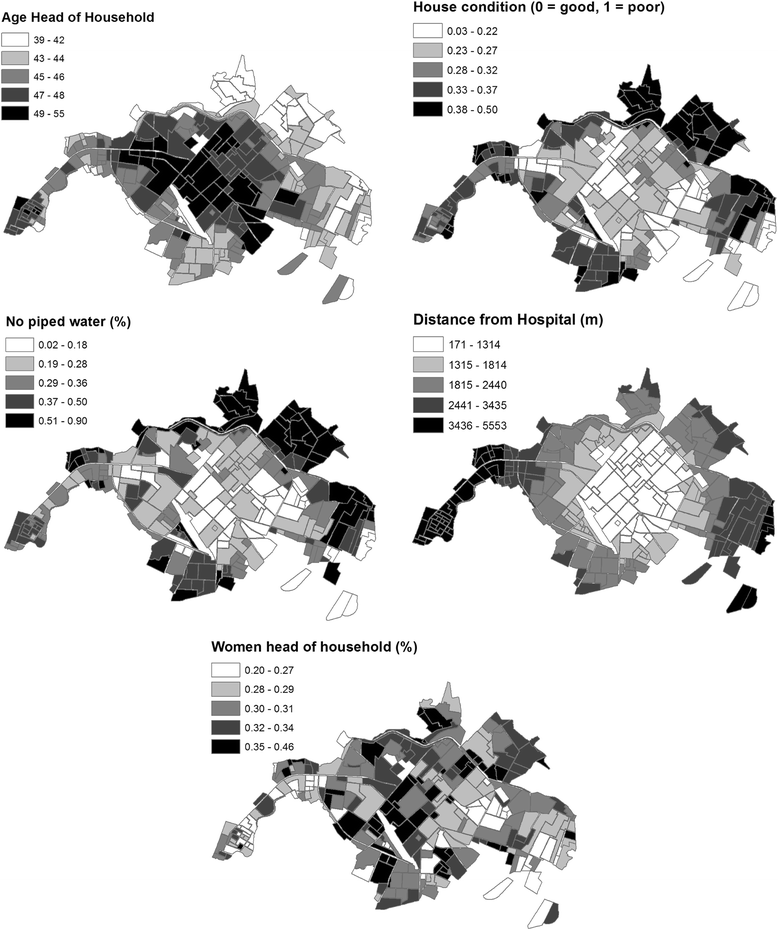

Supplement: Supplementary file 12 — Authors’ original file for figure 8 [file 12879_2014_610_MOESM12_ESM.gif]

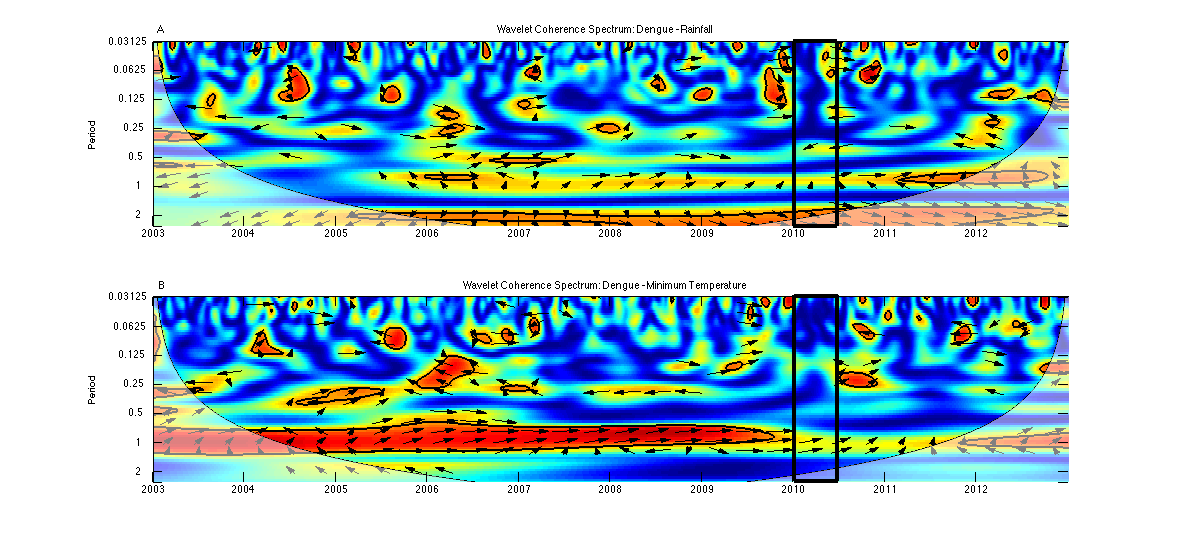

Supplement: Supplementary file 13 — Authors’ original file for figure 9 [file 12879_2014_610_MOESM13_ESM.tiff]
