# Supplementary material for: Spatiotemporal clustering, climate periodicity, and social-ecological risk factors for dengue during an outbreak in Machala, Ecuador, in 2010
Source: BMC Infect Dis. 2014 Nov 25;14:610. doi: 10.1186/s12879-014-0610-4 (PMC4264610; doi:10.1186/s12879-014-0610-4)
Supplement: Supplementary file 4 — Additional file 4: Table S2.: Top competing logistic regression models (ΔAICc < 2 or Weight > 1.5%) from multi-model selection to predict the presence (1) and absence (0) of dengue at the neighborhood level in Machala in 2010. (DOCX 16 KB) [file 12879_2014_610_MOESM4_ESM.docx]

| **Table S2. Top competing logistic regression models (ΔAICc < 2 or Weight > 1.5%) from multi-model selection to predict the presence (0) and absence (0) of dengue at the neighborhood level in Machala in 2010.*** | | | | |
| --- | --- | --- | --- | --- |
| # | Model | AICc | Weights | **∆**AICc |
| 1 | x ~ 1 + **wmhead** + headhh_age + **HCI_pipwat + dist_hosp** | 291.28 | 0.029 | 0.00 |
| 2 | x ~ 1 + **wmhead + headhh_age + HCI_pipwat + dist_hosp** + bi_12 | 291.87 | 0.020 | 0.59 |
| 3 | x ~ 1 + popdens + wmhead + headhh_age + **HCI_pipwat + dist_hosp** + bi_12 | 292.32 | 0.019 | 1.04 |
| 4 | x ~ 1 + popdens + wmhead + headhh_age + **HCI_pipwat + dist_hosp** | 292.22 | 0.018 | 0.95 |
| 5 | x ~ 1 + emigrt + wmhead + **HCI_pipwat + dist_hosp** | 292.39 | 0.017 | 1.12 |
| 6 | x ~ 1 + **wmhead + headhh_age** + pave + **HCI_pipwat + dist_hosp** | 292.62 | 0.017 | 1.35 |
| 7 | x ~ 1 + emigrt + wmhead + headhh_age + **HCI_pipwat + dist_hosp** | 292.49 | 0.016 | 1.21 |
| *Significant parameters (*P* ≤ 0.05) are bolded. | |  |  |  |

| **Parameter dictionary** | |
| --- | --- |
| wmhead | = Head of household is a woman (% households) |
| headhh_age | = Mean age of the head of the household |
| HCI_pipwat | = Housing condition index (HCI) regressed on households with no access to piped water insides the home |
| dist_hosp | = Average distance to the central hospital (km) |
| bi_12 | = Average Breteau Index during the first two quarters of 2010 |
| popdens | = Population density (people per square kilometer) |
| emigrt | = People emigrate for work (% households) |
| pave | = No access to paved roads (% households) |
